# Primärversorgungssystem in Deutschland: Evidenz, Chancen und Implementierung

**DOI:** 10.1007/s00103-026-04296-6

**Published:** 2026-09-17

**Authors:** Martin Scherer, Thomas Zimmermann, Eva Hummers

**Affiliations:** 1 Institut und Poliklinik für Allgemeinmedizin, Universitätsklinikum Hamburg-Eppendorf, Martinistraße 52, 20246 Hamburg, Deutschland; 2 Institut für Allgemeinmedizin, Universitätsmedizin Göttingen, Göttingen, Deutschland

**Keywords:** Primärversorgung, Hausarztzentrierte Versorgung, Versorgungskoordination, Gesundheitssystemvergleich, Leistungsindikator, Primary care, General practitioner-centered care, Care coordination, Healthcare system comparison, Performance indicator

## Abstract

Internationale Vergleiche zeigen, dass Gesundheitssysteme mit einer starken, koordinierenden Primärversorgung häufig bessere Gesundheitsoutcomes bei zugleich effizienterem Ressourceneinsatz erreichen. Vor diesem Hintergrund gibt dieser narrative Review auf Grundlage der verfügbaren Literatur sowie von Daten der Organisation für wirtschaftliche Zusammenarbeit und Entwicklung (OECD) einen Überblick über Strukturen, Inanspruchnahme und Outcomes der Gesundheitsversorgung in Deutschland im Vergleich zu 11 weiteren europäischen Ländern und ordnet die Befunde im Kontext hausärztlich koordinierter Versorgungssysteme ein.

Die Ergebnisse zeigen, dass Deutschland durch eine hohe Inanspruchnahme medizinischer Leistungen und hohe Gesundheitsausgaben gekennzeichnet ist, gleichzeitig jedoch bei zentralen Gesundheitsindikatoren, insbesondere der vermeidbaren Sterblichkeit, vergleichsweise ungünstig abschneidet. Eine stärkere Koordination durch die Primärversorgung könnte hier Verbesserungen ermöglichen.

Gleichzeitig verdeutlichen die Befunde, dass der Erfolg primärärztlich koordinierter Versorgungssysteme entscheidend von ihren strukturellen Rahmenbedingungen abhängt. Neben ausreichenden hausärztlichen Kapazitäten sind interprofessionelle Teams, leistungsfähige digitale Infrastrukturen sowie verbindliche Koordinations- und Rückkopplungsmechanismen zentrale Voraussetzungen. Eine stärkere Primärversorgung allein reicht jedoch nicht aus: Ihre Wirksamkeit setzt tiefgreifende Strukturreformen und eine stärkere Ausrichtung des Gesundheitssystems auf präventive Maßnahmen voraus.

## Abstract (en)

International comparisons show that healthcare systems with strong, coordinated primary care often achieve better health outcomes while using resources more efficiently. Against this background, this narrative review, based on the available literature and data from the Organisation for Economic Co-operation and Development (OECD), provides an overview of healthcare structures, utilization, and outcomes in Germany compared with 11 other European countries and interprets the findings in the context of healthcare systems coordinated by primary care.

The results show that Germany is characterized by high utilization of healthcare services and high healthcare expenditure, while at the same time performing comparatively poorly on key health indicators, particularly avoidable mortality. Stronger coordination through primary care could help improve these outcomes.

At the same time, the findings highlight that the success of primary care-coordinated healthcare systems depends critically on their structural framework. In addition to sufficient primary care capacity, interprofessional teams, robust digital infrastructure, and binding mechanisms for coordination and feedback are essential prerequisites. Strengthening primary care alone, however, is not sufficient: its effectiveness requires far-reaching structural reforms and a stronger orientation of the healthcare system toward preventive measures.

## Einleitung

Mit der Einführung eines Primärarztsystems in Deutschland [1] ist die Hoffnung verbunden, das überlastete Gesundheitssystem neu auszurichten sowie Versorgungsprozesse effektiver und effizienter zu gestalten. Daraus leitet sich die Frage ab, unter welchen strukturellen Voraussetzungen ein hausärztlich-koordiniertes Primärversorgungssystem im deutschen Kontext tragfähig wäre und welche Effekte davon zu erwarten sind. In dieser narrativen Übersicht verstehen wir darunter ein Versorgungssystem, in dem der Zugang zur Sekundärversorgung durch die Primärversorgung koordiniert und gesteuert wird. Zentrale Elemente sind Einschreibemodelle und die Koordination durch die hausärztliche Praxis. Ein solches System bietet einen gleichberechtigten Zugang aller Bevölkerungsgruppen zur gesundheitlichen Versorgung, erbracht durch hausärztlich geleitete interprofessionelle Teams [2, 3].

Die primäre Gesundheitsversorgung eines Landes ist die erste Schnittstelle zu einem System medizinisch-pflegerischer Leistungsangebote [4]. Der Zugang erfolgt niedrigschwellig, wohnort- und bedarfsnah. Die Weltgesundheitsorganisation (WHO; [5]) benennt über die medizinische Behandlung hinaus weitere Charakteristika: Priorisierung der wichtigsten Leistungen für Einzelpersonen und Familien durch die primärversorgende Einrichtung sowie den Einbezug sozialer, wirtschaftlicher und umweltbedingter Faktoren, aber auch patientenindividueller Merkmale.

In Deutschland stellen Hausärztinnen und Hausärzte die primärärztliche, kontinuierliche und umfassende Versorgung von Menschen mit allen Gesundheitsanlässen sicher. Körperliche, psychische und soziale Dimensionen von Gesundheit und Krankheit sowie deren Einbettung in Familie, Lebenswelt und Gesellschaft werden in Diagnostik, Therapie und Weiterbehandlung einbezogen. Die primärversorgenden Praxen stehen Menschen aller Altersstufen offen als wohnortnahe ärztliche Anlaufstelle mit niedrigschwelligem Zugang [6].

Vorläufer dieser Begriffsbestimmungen ist eine versorgungspraktische Perspektive [7, 8], die im Englischen meist kurz Primary Care genannt wird und durch folgende Merkmale gekennzeichnet ist:

- *erster Kontakt ins (Regel‑)Versorgungssystem*, damit Menschen bei Versorgungsbedarf eine ihnen bekannte Ansprechadresse haben;
- *Kontinuität in der (Regel‑)Versorgung*, die in Registerstudien [9, 10] und Reviews [11–14] überwiegend positive Effekte zeigt;
- *umfassende Versorgung* im Sinne fallabschließender Behandlung (bei ca. 80 % der Beratungsanlässe [15], bei 54,7 % der Akutfälle ohne weitere Inanspruchnahme innerhalb der folgenden 2 Wochen [16], bei ca. 90 % der Anlässe [17]). In einer weiteren Fallauswertung wurden 13,7 % der Fälle in die sekundäre, gebietsärztliche Versorgung überwiesen [18];
- *Koordination der gebietsärztlichen Versorgung*. Diese Steuerungsfunktion ist mit Kosteneffektivität assoziiert [19, 20]. Trotz guter Einzelstudien ist die Studienlage zur Verbesserung der Versorgungsqualität uneindeutig [21].

Derzeit werden in Deutschland zahlreiche Modelle mit mehr Primärversorgungsorientierung erprobt und durch Förderprogramme unterstützt, um neue Formen der Zusammenarbeit und Leistungserbringung jenseits üblicher Abrechnungsmodalitäten zu ermöglichen [22]. Zu den innovativsten zählen integrierte Versorgungszentren, kommunale oder freigemeinnützige medizinische Versorgungszentren sowie lokale Gesundheitszentren in Kooperation mit dem Öffentlichen Gesundheitsdienst. Ziel ist es, Aufgaben auf verschiedene Berufsgruppen (Physician Assistants, Advanced Practice Nurses, Versorgungsassistenzen in der Hausarztpraxis, Sozialarbeiter/Sozialarbeiterinnen) zu verteilen und ärztliche Ressourcen auf Kernaufgaben zu konzentrieren.

In Deutschland ist die ärztliche Versorgung durch einen offenen, d. h. ungesteuerten Zugang zu fast allen haus- und gebietsärztlichen Leistungen und Fachgebieten gekennzeichnet. Eine Differenzierung zwischen den Ebenen hausärztlicher und gebietsärztlicher Versorgung betrifft vor allem Abrechnungsmodalitäten und die Planung von Versorgungsstrukturen, weniger die Versorgungspfade aus Nutzer- oder Anbieterperspektive.

Ziel dieser Arbeit ist es, durch einen Vergleich von Struktur- und Leistungsindikatoren verschiedener OECD-Länder den Blick aus dem eigenen System heraus zu öffnen und Anregungen für Reformüberlegungen zu gewinnen. Dazu werden Gesundheitssysteme klassifiziert und zueinander in Bezug gesetzt. Der Vergleich von individuellen Systemkonfigurationen und die Einordnung hinsichtlich Einschreibebedingungen, Finanz- und weiteren systembezogenen Indikatoren der Leitstungserbringung sollen Möglichkeiten eröffnen, veränderte Versorgungskonzepte und -prozesse zu entwickeln sowie den Einsatz von Ressourcen zu überdenken.

## Ländervergleich: Quellen, Daten und Indikatoren

Für diese Arbeit vergleichen wir primärversorgungsrelevante Versorgungsstrukturen, finanz- und weitere systembezogene Indikatoren sowie ausgewählte Gesundheitsoutcomes in Deutschland mit denen von 11 ähnlich entwickelten europäischen Ländern. Wir beziehen uns einerseits auf die Vorarbeit von [23]. Dort wurden 10 Länder hinsichtlich primärärztlicher Versorgungsmerkmale verglichen. Andererseits nutzen wir die Klassifizierung von Böhm et al. (2013, [24]). Diese Autoren haben die Versorgungssysteme von 30 Ländern anhand der Dimensionen Regulation, Finanzierung und Leistungserbringung deduktiv verschiedenen Clustern zugeordnet.

Demgemäß vergleichen wir 12 Länder, deren Versorgungssysteme 4 Clustern zugeordnet werden:

1. *Sozialversicherungen*: Österreich (Länderkennung: AT); Schweiz (CH), Deutschland (DE);
2. *nationale Gesundheitsdienste*: Dänemark (DK), Spanien (ES), Finnland (FI), Norwegen (NO), Vereinigtes Königreich (UK);
3. *staatlich einheitliche Sozialversicherungen*: Frankreich (FR), Niederlande (NL), Polen (PL);
4. *nationale Gesundheitsversicherung*: Italien (IT).

Die Auswahl sollte an Wittlinger und Sundmacher [23] anschlussfähig sein, alle 4 Cluster von Böhm et al. [24] abbilden, Deutschland und Österreich einbeziehen und zugleich die innerhalb der Cluster bestehende Varianz berücksichtigen. Polen wurde als Transitions- und Transformationsland ergänzt, das trotz zunehmend verbesserter ökonomischer Rahmenbedingungen weiterhin eine vergleichsweise hohe, historisch geprägte Morbiditätslast aufweist.

Als Datenmaterial verwenden wir die Datenbank der OECD (https://data-explorer.oecd.org/) und eine Auswahl der Gesundheitsindikatoren, die dort hinterlegt sind und fortlaufend aktualisiert werden (Stand der Daten: 14.08.2026).

Zum einen entschieden wir uns für Gesundheitsoutcomes, die primärärztlich relevant sind: Anzahl vermeidbarer Krankenhauseinweisungen aufgrund häufiger chronischer Erkrankungen (Asthma bronchiale/chronisch obstruktive Lungenerkrankung (COPD), Diabetes mellitus, Herzinsuffizienz jeweils pro 100.000 Einwohner) sowie die Impfrate gegen Influenza (in Prozent) der Personen, die 65 Jahre und älter sind. Zum anderen bezogen wir die (durch Public-Health-Maßnahmen oder Primärprävention) verhinderbare und die (durch Maßnahmen der verbesserten Leistungserbringung) behandelbare Sterblichkeit sowie deren Summe, die sogenannte vermeidbare Sterblichkeit (jeweils Anzahl der Todesfälle pro 100.000 Einwohner), ein. Des Weiteren berücksichtigten wir die mittlere Lebenserwartung sowie die Differenz der mittleren Lebenserwartung zwischen Frauen und Männern in Jahren.

Als finanz- und systembezogene Indikatoren wählten wir Ausgaben für Medikamente pro Kopf der Bevölkerung (kaufkraftbereinigt in US-Dollar), Gesundheitsausgaben insgesamt pro Kopf (kaufkraftbereinigt in Euro), die persönliche und telemedizinische Inanspruchnahme von Ärztinnen und Ärzten (Anzahl Konsultationen pro Person pro Jahr), die Durchführung von Computertomographien und Magnetresonanztomographien (Anzahl pro 1000 Personen) und die Anzahl der im Indexjahr graduierten Ärztinnen und Ärzte (pro 100.000 Einwohner).

Primärversorgungsrelevante Befunde: In den hier verglichenen Ländern werden die Versicherten entweder automatisch registriert in einer wohnortnah gelegenen hausärztlichen Praxis (ES, FI, NO) oder sie schreiben sich freiwillig in eine Praxis ihrer Wahl ein (CH, DE, DK, UK, FR, NL, PL, IT). Nur in Österreich (AT) ist keinerlei Einschreibesystem vorgesehen (Tab. 1). Die resultierenden Einschreiberaten sind ebenfalls in Tab. 1 dargestellt.

**Tab. 1:** Eigenschaften von Primärversorgungssystemen in 12 OECD-Ländern

		Cluster 1			Cluster 2					Cluster 3			Cluster 4
	Land	AT	CH	DE	DK	ES	FI	NO	UK	FR	PL	NL	IT
*Ablauf der Registrierung*	Automatisch	–*	–	–	–	x	x	x	–	–	–	–	–
	Patienteninitiiert	–	x	x	x	–	–	–	x	x	x	x	x
	Registrierungsquote (geschätzt)	–	60 %	15 %	98 %	100 %	100 %	> 99 %	100 %	90–95 %	100 %	95 %	100 %
*Anreizwirkungen zur Teilnahme am Primärarztsystem*	Registrierung Voraussetzung für Inanspruchnahme	–	–	–	–	x	x	x	x	–	–	–	x
	Nachteile bei Terminvergabe ohne Registrierung	–	–	–	–	–	–	–	–	x	–	x	–
	Überweisung Voraussetzung für Inanspruchnahme weiterer Fachgebiete	–	–	–	–	x	x	x	x	–	–	–	x
	Höhere Zuzahlung ohne Überweisung	–	–	–	x	–	–	–	–	x	–	x	–
	Prämienersparnis bei Hausarztbindung	–	x	–	–	–	–	–	–	–	–	–	–
* Risikoabsicherung*	Automatische Deckung	–	–	–	x	x	x	x	x	–	x	–	x
	Pflichtversicherung	x	x	x	–	–	–	–	–	x	–	x	–

Cluster 1: Sozialversicherungen, Cluster 2: nationale Gesundheitsdienste, Cluster 3: staatlich einheitliche Sozialversicherungen, Cluster 4: nationale Gesundheitsversicherung

*AT* Österreich, *CH* Schweiz, *DE* Deutschland, *DK* Dänemark, *ES* Spanien, *FI* Finnland, *NO* Norwegen, *UK* Vereinigtes Königreich, *FR* Frankreich, *PL* Polen, *NL* Niederlande, *IT* Italien

Daten für CH, DE, DK, ES, FI, NO, UK, FR, NL, IT aus [23], ergänzt um eigene Recherche zu AT, DE, PL

* Österreich hat weder ein freiwilliges noch ein verpflichtendes Einschreibesystem

Die Einschreiberate in die freiwillige hausarztzentrierte Versorgung (HZV) in Deutschland ist mit 15 % der Gesamtbevölkerung vergleichsweise gering. Tab. 2 zeigt gesundheitsbezogene Indikatoren aus dem OECD-Datenpool. So weist Deutschland eine geringe Influenza-Impfrate bei Personen auf, die 65 Jahre oder älter sind, und liegt auf dem viertletzten Platz, unterboten nur von Österreich, der Schweiz und Polen.

**Tab. 2:** Gesundheitsbezogene Daten in 12 Ländern (Quelle: OECD Health Data, Stand 14.08.2026)

	Cluster 1			Cluster 2					Cluster 3			Cluster 4
Indikator/Land	AT	CH	DE	DK	ES	FI	NO	UK	FR	PL	NL	IT
Influenza-Impfrate in Prozent der Bevölkerung ≥ 65 Jahre (2023)	23,5 (2024)	37,5 (2022)	40,4	77,5	67 (2024)	58 (2024)	64,2	77,8	54 (2024)	9,6	66,5	56,7
Krankenhauseinweisungen wegen Asthma oder COPD pro 100.000 Einwohner (2023)	155,1	161,2	250,1	278,4	202,7	115,1	206,8	254,2	k. A.	127,6	125 (2021)	31,3
Krankenhauseinweisungen wegen Herzinsuffizienz pro 100.000 Einwohner (2023)	175,7	215,5	380,3	221,0	161,4	211,3	191,7	110,9	k. A.	520,9	152,4	162,3
Krankenhauseinweisungen wegen Diabetes mellitus pro 100.000 Einwohner (2023)	109,3	82,6	179,5	89,8	62,0	84,9	59,6	82,1	k. A.	160,1	42,3	30,7
Vermeidbare Sterblichkeit insgesamt (Todesfälle pro 100.000 Einwohner; 2024)	171,0	116,0 (2023)	192 (2022)	175,0 (2022)	136,0	170,0	k. A.	200	149,0 (2023)	272,0	144,0	133,0 (2023)
Verhinderbare Sterblichkeit (Todesfälle pro 100.000 Einwohner; 2024)	118,0	81,0 (2023)	129 (2022)	120,0 (2022)	188,0	117,0	k. A.	127	103,0 (2023)	166,0	97,0	82,0
Behandelbare Sterblichkeit (Todesfälle pro 100.000 Einwohner; 2024)	53,0	35,0 (2023)	62 (2022)	55,0 (2022)	48,0	53,0	k. A.	73	46,0 (2023)	106,0	47,0	51,0
Lebenserwartung bei Geburt in Jahren (2024)	82,1	84,2	81,2	82,0	84,0	82,2	83,2	81,3	83,0	78,5	81,9	83,7
Differenz der Lebenserwartung (Frauen – Männer) in Jahren (2024)	4,5	3,5	4,6	3,7	5,1	5,2	3,2	3,9	5,6	7,4	2,8	4,1

Cluster 1: Sozialversicherungen, Cluster 2: nationale Gesundheitsdienste, Cluster 3: staatlich einheitliche Sozialversicherungen, Cluster 4: nationale Gesundheitsversicherung

*AT* Österreich, *CH* Schweiz, *DE* Deutschland, *DK* Dänemark, *ES* Spanien, *FI* Finnland, *NO* Norwegen, *UK* Vereinigtes Königreich, *FR* Frankreich, *PL* Polen, *NL* Niederlande, *IT* Italien

*k.A.* keine Angabe; einzelne Jahresangaben in den Zellen zeigen den letzten verfügbaren Wert des jeweiligen Indikators

Bei potenziell vermeidbaren Krankenhauseinweisungen (aufgrund von Asthma/COPD, Herzinsuffizienz, Diabetes mellitus) gehört Deutschland durchweg zu den Versorgungssystemen mit den höchsten Einweisungsraten. Aufgrund von Herzinsuffizienz wird in Deutschland 3,5-mal häufiger eingewiesen als im Land mit der geringsten Rate, dem Vereinigten Königreich. Bei diabetesbedingten Krankenhauseinweisungen weist Deutschland mit 179,5 Fällen pro 100.000 Einwohner den höchsten Wert aller verglichenen Länder auf. Dieser liegt 6‑mal so hoch wie der niedrigste Wert aus Italien.

Auch bei der vermeidbaren Sterblichkeit zählt Deutschland zu den 3 Ländern mit den höchsten Werten, nach Polen und dem Vereinigten Königreich. Ein ähnliches Bild zeigt sich bei der Lebenserwartung: Mit 81,2 Jahren liegt Deutschland nur knapp vor dem Vereinigten Königreich (81,3 Jahre) als Zweitletztem. Nur in Polen ist die Lebenserwartung bei Geburt 2024 noch geringer (78,5 Jahre). Bei der Differenz der mittleren Lebenserwartung zwischen Frauen und Männern liegt Deutschland dagegen im Mittelfeld. In Polen überleben Frauen die Männer im Schnitt 7,5 Jahre, in Finnland, Frankreich und Spanien sind es jeweils etwas mehr als 5 Jahre.

Die Inanspruchnahme von Ärztinnen und Ärzten in Deutschland ist vergleichsweise hoch (9,7 persönliche Kontakte pro Person pro Jahr) und wird nur von den Niederlanden übertroffen, wo es zu 10,6 persönlichen Kontakten kommt (Tab. 3).

**Tab. 3:** System- und finanzbezogene Indikatoren in 12 Ländern (Quelle: OECD Health Data, Stand 14.08.2026)

	Cluster 1			Cluster 2					Cluster 3			Cluster 4
Indikator/Land	AT	CH	DE	DK	ES	FI	NO	UK	FR	PL	NL	IT
Persönliche Inanspruchnahme Ärztinnen und Ärzte (pro Jahr; 2024)	k. A.	4,5 (2022)	9,7	5	6,1	3,2	4	k. A.	5,3	8,1	10,6	9,4 (2023)
Telemedizinische Inanspruchnahme Ärztinnen und Ärzte (pro Jahr; 2024)	k. A.	k. A.	0,1	1,6	1,5	1,0	0,7	k. A.	0,2	0,7	k. A.	k. A.
Computertomographie (Prozeduren pro 1000 Personen; 2024)	226,2 (2023)	k. A.	176,3	203,7	154,3	95	92,9	k. A.	241,4	161,4	138,5	116,5
Magnetresonanztomographie (Prozeduren pro 1000 Personen; 2024)	168,1 (2023)	k. A.	166,6	89	130,5	70,2	133	k. A.	162,9	72,8	59,9	84,3
Ausgaben für Medikamente in US-Dollar pro Person (kaufkraftbereinigt; 2024)	707,8	941,9	1013	861	951,3	736,8	813,2	818,2	609,9	255,8	432,9	791,1
Gesundheitsausgaben in Euro pro Person (kaufkraftbereinigt; 2024)	5347	6232	5790	4471	3232	4100	5585	4348	4491	2615	5254	3225
Ausgebildete Ärztinnen und Ärzte (Anzahl pro 100.000 Einwohner; 2024)	17,6	15,3	12,4	21,8	13,6	13,4	11,2	13,5 (2022)	11,0	16,3	14,3	17,0

Cluster 1: Sozialversicherungen, Cluster 2: nationale Gesundheitsdienste, Cluster 3: staatlich einheitliche Sozialversicherungen, Cluster 4: nationale Gesundheitsversicherung

*AT* Österreich, *CH* Schweiz, *DE* Deutschland, *DK* Dänemark, *ES* Spanien, *FI* Finnland, *NO* Norwegen, *UK* Vereinigtes Königreich, *FR* Frankreich, *PL* Polen, *NL* Niederlande, *IT* Italien

*k.* *A.* keine Angabe; einzelne Jahresangaben in den Zellen zeigen den letzten verfügbaren Wert des jeweiligen Indikators

Deutschland weist nach Österreich die zweithöchste Zahl an Magnetresonanztomographien auf. Beim Einsatz der Computertomographie liegt Deutschland an vierter Stelle hinter Frankreich, Österreich und Dänemark. Für die Schweiz sind hierzu keine vergleichbaren Daten verfügbar. Ebenfalls hoch sind die deutschen Ausgaben für Medikamente und für Gesundheit insgesamt: Neben den höchsten Pro-Kopf-Ausgaben für Medikamente (kaufkraftbereinigt 1013 US $ im Jahr 2024) weist Deutschland mit 5790 € die zweithöchsten Gesundheitsausgaben pro Kopf auf, nach der Schweiz (6232 €) und vor Norwegen (5585 €).

Betrachtet man die Indikatoren zu den Krankenhauseinweisungen zu 2 Zeitpunkten (2015; 2023), ist der allgemeine Trend zu stärkerer Ambulantisierung in fast allen Versorgungssystemen erkennbar. Doch gelang es anderen Gesundheitssystemen deutlich besser als dem deutschen, die Zahl der Einweisungen zu reduzieren (Tab. 4). Bei ähnlicher Ausgangslage ist es der Niederlande beispielsweise gelungen, die Einweisungsraten aufgrund von Asthma/COPD innerhalb von nur 6 Jahren zu halbieren (−52,4 %), Österreich erreichte eine Reduktion von 44,3 %. In Deutschland betrug der Rückgang lediglich 17,2 %.

**Tab. 4:** Krankenhauseinweisungen zu 2 Zeitpunkten (2015; 2023) in 12 Ländern (Quelle: OECD Health Data, Stand 14.08.2026)

		Cluster 1			Cluster 2					Cluster 3			Cluster 4
Indikator	Jahr	AT	CH	DE	DK	ES	FI	NO	UK	FR	NL	PL	IT
Krankenhauseinweisungen wegen Asthma oder COPD pro 100.000 Einwohner	2015	279,5	176,5	301,9	386,3	249,9	183,2	279,0	319,8	158,6	262,6	247,4	68,7
	2023	155,1	161,2	250,1	278,4	202,7	115,1	206,8	254,2	k. A.	125 (2021)	127,6	31,3
Krankenhauseinweisungen wegen Herzinsuffizienz pro 100.000 Einwohner	2015	275,0	211,1	416,1	331,3	210,5	284,9	172,0	108,3	287,0	165,9	486,3	248,8
	2023	175,7	215,5	380,3	221,0	161,4	211,3	191,7	110,9	k. A.	152,4	520,9	162,3
Krankenhauseinweisungen wegen Diabetes mellitus pro 100.000 Einwohner	2015	203,9	80,0	228,0	142,5	48,6	119,2	74,8	73,2	155,6	62,6	206,5	54,0
	2023	109,5	82,6	179,5	89,8	62,0	84,9	59,6	82,1	k. A.	42,3	160,1	30,7

Cluster 1: Sozialversicherungen, Cluster 2: nationale Gesundheitsdienste, Cluster 3: staatlich einheitliche Sozialversicherungen, Cluster 4: nationale Gesundheitsversicherung

*AT* Österreich, *CH* Schweiz, *DE* Deutschland, *DK* Dänemark, *ES* Spanien, *FI* Finnland, *NO* Norwegen, *UK* Vereinigtes Königreich, *FR* Frankreich, *PL* Polen, *NL* Niederlande, *IT* Italien

*k.* *A.* keine Angabe; einzelne Jahresangaben in den Zellen zeigen den letzten verfügbaren Wert des jeweiligen Indikators

Bei Einweisungen wegen Herzinsuffizienz blieb Deutschland über 8 Jahre auf hohem Niveau, zu beiden Zeitpunkten nur von Polen übertroffen. Der Rückgang zwischen den beiden Zeitpunkten war mit 8,6 % deutlich geringer als in Dänemark (33,2 %) oder Österreich (36,4 %). Bei Diabetes mellitus konnte Österreich die Einweisungen nahezu halbieren (46,3 %), Deutschland erreichte lediglich eine Reduktion von 27 %, Polen etwa 20 %.

## Deutschland im Vergleich: Notwendigkeit der Weiterentwicklung von Versorgungsstrukturen

Insgesamt zeigt sich, dass die Leistungsindikatoren in Deutschland trotz hoher Inanspruchnahme und hoher Ausgaben eher unterdurchschnittlich ausfallen. Dieser bereits bekannte Befund wird durch die aktuellen OECD-Daten erneut bestätigt. Günstigere Ergebnisse finden sich sowohl in steuerfinanzierten als auch in Gesundheitssystemen mit einheitlicher Sozialversicherung. Gemeinsam ist diesen Ländern mit vergleichsweise besseren Ergebnissen eine stärkere Rolle der Primärversorgung bei der Steuerung von Versorgungspfaden. Die dargestellten Daten sprechen dafür, dass eine stärkere primärärztliche Koordination ein möglicher Ansatz zur Weiterentwicklung bestehender Versorgungsstrukturen sein könnte.

Es wäre jedoch wissenschaftlich nicht belastbar, einen kausalen Zusammenhang mit der Abwesenheit eines verpflichtenden Primärversorgungssystems zu erklären. Der Ländervergleich deutet zudem darauf hin, dass Unterschiede in den Leistungsindikatoren nicht allein durch die Organisation der medizinischen Versorgung erklärt werden können. Besonders auffällig ist, dass Länder mit günstigen Indikatoren häufig zugleich eine stärkere Public-Health-Orientierung aufweisen. So schneiden beispielsweise die Niederlande sowohl bei mehreren Indikatoren als auch im Bereich bevölkerungsbezogener Präventionsstrategien besser ab als Deutschland. Dies spricht dafür, dass Verbesserungen bei Leistungsindikatoren nicht allein durch Veränderungen der Versorgungssteuerung erreicht werden können.

So gibt es zentrale Handlungsfelder für eine gesündere Gesellschaft: Ernährung (z. B. Zuckerreduktion), Alkohol- und Tabakkonsum (z. B. Werbebeschränkungen, besserer Nichtraucherschutz) sowie körperliche Aktivität (z. B. Infrastruktur für Bewegungsförderung; [25]). Der Schlüssel für eine gesündere Gesellschaft liegt weit vor der Praxis- und Kliniktür. Entscheidender ist die Verschränkung von Verhaltens- und Verhältnisprävention: Lebensumstände und Alltagsbedingungen müssen so gestaltet sein, dass gesundes Verhalten erleichtert wird.

Entsprechend schneiden Länder, die in diesen Bereichen der Verhältnisprävention weiter entwickelt sind als Deutschland (Tab. 4), auch bei Leistungsindikatoren besser ab (z. B. die Niederlande [26], Tab. 4). Interessant ist in diesem Zusammenhang, dass die 3 sozialversicherungsbasierten Versorgungssysteme in unserer Auswahl (Österreich, Schweiz und Deutschland) auf dem „Public Health Index (PHI)“ [27] die 3 hintersten Plätze belegen. Die staatlichen Systeme im Vereinigten Königreich, Finnland und Norwegen hingegen platzieren sich auf 1, 3 und 4. Eine Verbesserung gesundheitsbezogener Outcomes in Deutschland kann nur gelingen, wenn insbesondere das gesundheitliche und gesundheitsökonomische Potenzial der Verhältnisprävention stärker genutzt wird. Gleichzeitig reicht es nicht aus, allein die Lebensverhältnisse zu adressieren. Auch die Versorgungsstrukturen selbst müssen weiterentwickelt werden.

## Chancen und Herausforderungen einer Primärversorgung in Deutschland

Hier setzt die hausärztliche Primärversorgung an: Sie kann durch eine kontinuierliche und überwiegend fallabschließende Betreuung, die Priorisierung von Behandlungsbedarfen sowie eine koordinierte Zugangssteuerung dazu beitragen, unnötige Inanspruchnahmen zu reduzieren und Ressourcen zielgerichteter einzusetzen. Gesundheitssysteme mit Gatekeeping-Funktion weisen geringere Gesundheitsausgaben und eine höhere Versorgungsqualität, aber auch geringere Patientenzufriedenheit auf [28].

Die in vielen Ländern unserer vergleichenden Übersicht erreichten hohen Registrierungsquoten und Steuerungsfunktionen beruhen nicht allein auf der Tätigkeit einzelner Hausärztinnen und Hausärzte, sondern auf Anreizstrukturen (Inanspruchnahme oder bevorzugte Terminvergabe setzt Registrierung voraus; Überweisung erforderlich, um andere Fachgebiete einzubeziehen – Tab. 1) und häufig auf dem Einsatz multiprofessioneller Primärversorgungsteams.

In jüngerer Zeit wird die Rolle der hausärztlichen Primärversorgung zunehmend im Kontext multiprofessioneller Teammodelle diskutiert. Die Integration nichtärztlicher Gesundheitsberufe, insbesondere von Advanced Practice Nurses (APN), wird international als Strategie zur Bewältigung steigenden Versorgungsbedarfs und hausärztlicher Kapazitätsengpässe beschrieben. Erfahrungen aus OECD-Ländern deuten darauf hin, dass solche Modelle die Zugänglichkeit der Versorgung verbessern können [29, 30]. Ihre Wirksamkeit hängt jedoch stark von den strukturellen Rahmenbedingungen ab. Dazu zählen klare Regelungen und Zuständigkeiten, ausreichende hausärztliche Kapazitäten sowie multiprofessionelle Teams, die beispielsweise speziell weitergebildete medizinische Fachangestellte wie Versorgungsassistentinnen und -assistenten (VeraH) oder nichtärztliche Praxisassistentinnen und -assistenten (NäPa), akademische Gesundheitsberufe wie Primary Care Manager (PCM), Physician Assistants (PA) sowie spezialisierte Pflegekräfte wie Advanced Practice Nurses und Community Health Nurses umfassen [29].

Wesentlich ist daher, wie die in anderen Ländern beobachteten Koordinationsfunktionen innerhalb des deutschen Systems umgesetzt werden könnten. Während viele Vergleichsländer eine nahezu vollständige Registrierung der Bevölkerung in primärversorgenden Strukturen aufweisen, liegt die Einschreiberate in die freiwillige hausarztzentrierte Versorgung in Deutschland (HZV) derzeit nur bei etwa 15 %. Erfahrungen mit diesem Versorgungsmodell deuten auf eine verbesserte Schnittstellenkoordination, eine höhere Versorgungskontinuität sowie Verbesserungen ausgewählter Leistungsindikatoren hin [31, 32]. Delegation und eine veränderte Aufgabenverteilung können zudem dazu beitragen, hausärztliche Kapazitätsengpässe zu adressieren [33].

Die HZV stellt in Deutschland das bislang am weitesten entwickelte institutionelle Modell einer formal koordinierenden Primärversorgung dar. Die Basis ist eine auf Freiwilligkeit basierende vertragliche Selbstverpflichtung, vor der Inanspruchnahme der nächsthöheren Versorgungsebene zunächst die hausärztliche Versorgung aufzusuchen. Je konsequenter sich Versicherte daran halten, desto stärkere Effekte werden sichtbar, bspw. bei der Vermeidung stationärer Aufnahmen oder potenziell inadäquater Medikation [34]. Die konzeptuelle Weiterentwicklung der HZV zu sogenannten HÄPPI-Praxen (Hausärztliches Primärversorgungszentrum – Patientenversorgung Interprofessionell) strebt an, aus der Hausärzteschaft heraus die Grundprinzipien der Primärversorgung (Erstkontakt, Koordination, Kontinuität, Patientenorientierung, interprofessionelle Kooperation) systematisch im gegenwärtigen Versorgungssystem zu etablieren [35].

International gilt eine starke, die weitere Versorgung steuernde Primärversorgung als zentrales Element leistungsfähiger Gesundheitssysteme. Trotz der beschriebenen Potenziale ist die Einführung eines flächendeckenden Primärversorgungssystems mit erheblichen strukturellen Herausforderungen verbunden. Modelle mit unklaren Zuständigkeiten, parallel angelegten Versorgungswegen, mangelnder Integration in bestehende Versorgungsstrukturen oder unzureichender Finanzierung zeigen deutlich geringere Effekte als strukturell gut eingebettete Modelle [21, 28, 36].

Ein weiterer Diskussionspunkt betrifft die Patientenperspektive. Koordinierende Primärversorgungssysteme schränken die freie Arztwahl ein. Dies wird dadurch verschärft, dass spezialisierte Leistungen von Bürgerinnen und Bürgern nicht selten als „höherwertig“ wahrgenommen werden. Die Akzeptanz von Primärversorgungssystemen hängt stark von Transparenz, Zugänglichkeit sowie wahrgenommener und tatsächlicher Versorgungsqualität ab.

Schließlich stellt eine gut funktionierende digitale Infrastruktur eine zentrale Implementationsvoraussetzung dar. Ohne interoperable IT-Strukturen besteht die Gefahr zusätzlicher Bürokratie statt einer verbesserten Versorgungsintegration. Internationale Reformprozesse zeigen, dass Primärversorgungssysteme eng mit Digitalisierungsstrategien gekoppelt sein müssen, um ihre Steuerungsfunktion nutzbringend entfalten zu können. Entscheidend ist dabei eine gemeinsame, von allen an der Versorgung beteiligten Personen zugängliche elektronische Patientenakte mit vollständigen Befunden. Ebenso entscheidend sind verbindliche Rückkopplungsmechanismen zwischen den Versorgungsebenen. Eine spezialisierte Mitversorgung kann über längere Zeit notwendig sein, sollte jedoch nicht zu einer dauerhaften Verlagerung der Koordinationsfunktion von der Primär- in die Sekundärversorgung führen. Ein System, in dem Orthopädie, Kardiologie oder Urologie die hausärztliche Rolle übernehmen können, untergräbt die Koordinationsfunktion der Primärversorgung und gefährdet deren Qualität. Erforderlich sind daher klare Rückführungsmechanismen in die primärärztliche Betreuung, um Versorgungskontinuität zu sichern und Fragmentierung zu vermeiden.

## Fazit

Die dargestellten Befunde sprechen dafür, dass eine stärkere primärärztliche Koordination ein vielversprechender Ansatz zur Weiterentwicklung bestehender Versorgungsstrukturen sein könnte. Primärversorgungssysteme zeigen insbesondere bei chronisch kranken und multimorbiden Patientengruppen Potenziale zur Reduktion vermeidbarer Krankenhauseinweisungen und zur Stabilisierung ambulanter Versorgungsstrukturen. Welche Anreize gesetzt werden sollten, um die Registrierungsraten in einem Primärversorgungssystem im Vergleich zur HZV deutlich zu steigern, muss im Rahmen der notwendigen Gesetzesinitiative diskutiert werden.

## Data Availability

Alle dieser Arbeit zugrunde liegenden Daten sind in diesem Artikel enthalten. Diese und weitere Daten sind verfügbar unter https://data-explorer.oecd.org.
